# Electromechanical Response of Smart Ultra-High Performance Concrete under External Loads Corresponding to Different Electrical Measurements

**DOI:** 10.3390/s21041281

**Published:** 2021-02-11

**Authors:** Min Kyoung Kim, Huy Viet Le, Dong Joo Kim

**Affiliations:** 1 Department of Civil and Environmental Engineering, SEJONG University, 209, Neungdong-ro, Gwangjin-gu, Seoul 05006, Korea; mkkim9112@sejong.ac.kr (M.K.K.); lehuyviet@humg.edu.vn (H.V.L.); 2 Department of Civil Engineering, Hanoi University of Mining and Geology, Hanoi 100000, Vietnam

**Keywords:** smart materials, damage mechanics, self-sensing mechanism

## Abstract

This study investigated the electromechanical response of smart ultra-high-performance concretes (smart UHPCs), containing fine steel slag aggregates (FSSAs) and steel fibers as functional fillers, under external loads corresponding to different measurement methods. Regardless of different measurement methods of electrical resistance, the smart UHPCs under compression showed a clear reduction in their electrical resistivity. However, under tension, their electrical resistivity measured from direct current (DC) measurement decreased, whereas that from alternating current (AC) measurement increased. This was because the electrical resistivity, from DC measurement, of smart UHPCs was primarily dependent on fiber crack bridging, whereas that from AC measurement was dependent on tunneling effects.

## 1. Introduction

Smart construction materials (SCMs) with self-sensing capacity have great potential in the field of structural health monitoring (SHM) systems for civil infrastructures and buildings [1]. Much research has been extensively conducted to retain the self-sensing capacity of SCMs, even after initial cracking, by adding short steel and/or polyvinyl alcohol (PVA) fibers to mortar matrices under tension [2,3,4,5]. Strain-hardening cementitious composites (SHCCs), types of SCM containing short steel and/or PVA fiber, as functional fillers, have clearly demonstrated self-sensing capacity in the tensile strain-hardening region and generated multiple microcracks [4,5]. Both direct current (DC) and alternative current (AC) measurement methods have been used to investigate the electromechanical responses of strain-hardening steel-fiber-reinforced cementitious composites (SH-SFRCs) and engineered cementitious composites (ECCs) [5,6,7,8].

The electrical resistance (or real impedance) of SH-SFRCs decreased [2,3,4,5,6,7], whereas that of ECCs with polyvinyl alcohol (PVA) fibers increased as the tensile strain of them increased [5,9]. The difference in the electromechanical responses of SH-SFRCs and ECCs was based on the type of fiber. In addition, the reported electromechanical responses were measured from different (DC or AC) measurement methods [3,5]. The DC measurement delivers constant electrical currents to SCMs, but it requires considerable time to stabilize electrical polarization. On the other hands, AC measurement does not require considerable polarization time; thus, it quickly measures the electrical impedance of SCMs [1,10]. Although there are many studies in the literature reporting the difference between DC and AC measurements, it is difficult to find a suitable reference directly comparing the electrical resistance (or impedance) of SCMs corresponding to different DC and AC measurements.

Carbon black, granulated blast furnace slag, milled glass fibers, and fine steel slag aggregates have been utilized as additional fillers to further enhance the self-sensing capacity of SH-SFRCs [7,11,12,13]. The SH-SFRCs containing fine steel slag aggregates (FSSAs) instead of silica sand (i.e., smart ultra-high-performance concretes (smart UHPCs)) have demonstrated noticeably enhanced stress self-sensing capacity under compression [8]. However, the influence of different electrical (DC and AC) currents on the measured electromechanical responses of smart UHPCs under external loads, has not been investigated yet. In other words, it is not clear if the electromechanical responses of SCMs measured using DC measurement would be identical to those obtained using AC measurement. The electromechanical responses of SCMs under external loads should be investigated and compared under different current sources to find a suitable measurement method for self-strain-, -stress-, and -damage-sensing capabilities of SCMs, because DC and AC have different electron movement.

The self-sensing mechanisms of SCMs containing the functional fillers under external loads can be classified corresponding to the type of functional fillers (particle- or fiber- type). The SCMs containing particle-type functional fillers (e.g., nickel powder, carbon black, and steel slag aggregates) have exhibited sensing mechanisms that are primarily based on a conductive network or tunneling effect, regardless of the current sources [8,14,15]. On the other hand, the self-sensing mechanisms of SCMs containing fiber-type functional fillers (e.g., Ni nanowire, carbon, PVA, and steel fibers) are dependent upon loading conditions [3,5,16,17]. Under compression, the self-sensing mechanisms of SCMs containing fiber-type functional fillers are similar to that of those containing particle-type functional fillers [8,17,18]. However, under tension, the self-sensing mechanisms of SCMs were found to be different and closely related to the electrical resistance of their bonded and debonded fiber-matrix interfaces during fiber pullout [19,20,21].

To clarify self-strain-, -damage-, and -stress-sensing mechanisms, as well as to find a more suitable method for the measurement of electromechanical response of smart UHPCs under specific loading conditions, in this study, we investigated the electromechanical responses of smart UHPCs, containing steel fibers and FSSAs as functional fillers, corresponding to different electrical currents (DC or AC) under external loads. The detailed objectives of this study were: (1) to investigate the electromechanical responses of smart UHPCs corresponding to the current sources (DC or AC); (2) to evaluate the self-strain, -damage, and -stress-sensing capacity of smart UHPCs; and (3) to understand the sources of different electromechanical responses of smart UHPCs under external loads corresponding to different (DC or AC) measurement methods.

## 2. Experimental

**Figure 1:**
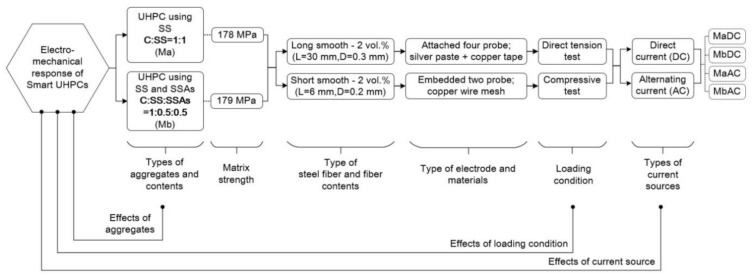
Experimental program.

**Figure 2:**
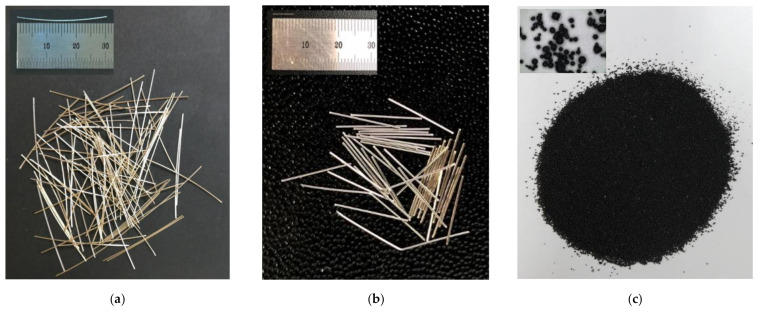
Geometry of functional fillers: (**a**) long smooth steel fibers, (**b**) short smooth steel fiber, (**c**) fine steel slag aggregates.

Figure 1 illustrates the experimental program designed to investigate the electromechanical responses of smart UHPCs corresponding to different current sources under various loading conditions. Table 1 shows the compositions of the matrices used in the experimental program: Ma is a typical matrix composition using silica sand, whereas Mb replaced 50% of silica sand with fine steel slag aggregates (FSSAs). Table 2 provides the properties of functional fillers, including steel fibers and FSSAs. The length and diameter of steel fibers used for tensile specimens were 30 and 0.3 mm (long smooth), respectively, whereas those for compressive specimens were 6 and 0.2 mm (short smooth), respectively. The content of steel fibers added to the three matrices was 2 vol.% for all specimens. Figure 2 shows the images of functional fillers, including steel fibers and FSSAs, used in the experimental program. The electrical response (resistance or impedance) of smart UHPCs was measured using both direct current (DC) and alternating current (AC) under direct tension and compression.

### 2.1. Materials and Specimen Preparation

Table 1 provided the composition and compressive strength of the two mortar matrices, while Table 2 summarized the properties of the steel fibers (long and short smooth) and FSSAs. The components of all mortar matrices were type 1 cement, silica fume, silica powder, and silica sand. FSSAs were added to only Mb as a partial replacement of silica sands. The average grain diameter of silica sand in all mortar matrices was 0.30 mm. The maximum diameter of ball-shaped FSSAs was 0.39 mm. The compressive strengths of Ma and Mb were measured as 178 and 179 MPa, respectively [12].

A Hobart-type laboratory mixer with a capacity of 20 L was used for mortar mixing. The components of the matrices (including the cement, silica fume, silica powder, and silica sand or/and FSSAs) were first dry-mixed for 5 min, then water was added to the mixture, and the mortar mixture was mixed for 5 min. The superplasticizer was gradually added, followed by further mixing for 5 min. When the workability of mortar matrices was suitable for uniform fiber distribution, then steel fibers were carefully dispersed by hand into the mortar mixtures, and the mixture containing fibers was then mixed for 1 min.

**Figure 3:**
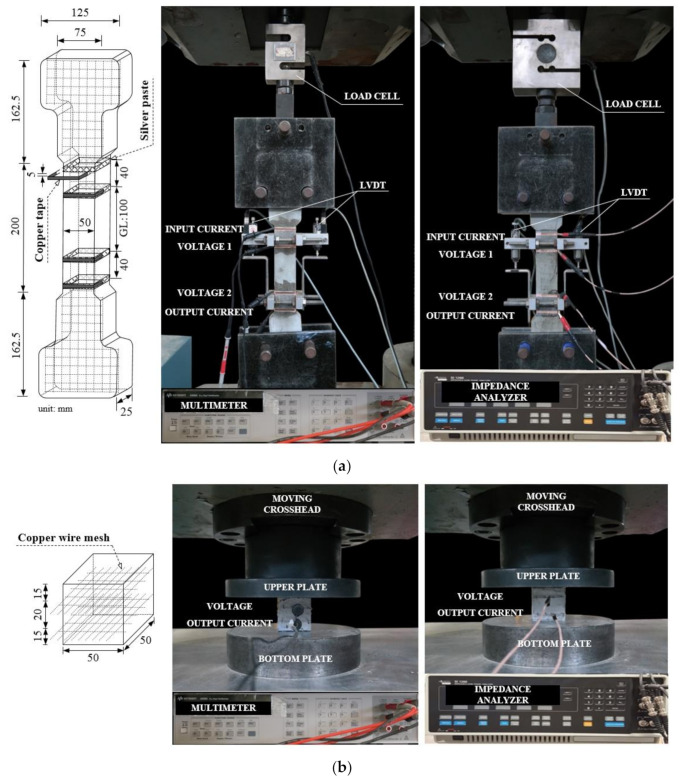
Test set-up: (**a**) direct tensile test under direct current (DC) or alternating current (AC) measurement, (**b**) compressive test under direct current (DC) or alternating current (AC) measurements.

The gauge length of dumbbell-shaped tensile specimens was 100 mm, while the cross-sectional area of the specimens was 25 mm × 50 mm, as shown in Figure 3a. Two layers of steel wire mesh were reinforced at both ends of the specimen to prevent failure outside the gauge length, as shown in Figure 3a. The cubic specimens (50 mm × 50 mm × 50 mm) were prepared, as shown in Figure 3b, to investigate the electromechanical responses of smart UHPCs under compression.

When the mortar mixture showed a suitable workability, steel fibers were added to the mixture and then further mixed for 1 min. The mortar mixture with fibers was then poured into molds. After casting, all specimens were covered with plastic sheets and placed in a laboratory at room temperature (25 °C) and 60% relative humidity for 48 h prior to demolding. After demolding, the specimens were water-cured at 90 °C for three days.

For AC measurement, the tensile specimen was electrically grounded from the steel grip by coating the grip region with epoxy to insulate the electrodes. A copper tape electrode was mounted on the surface of the cured specimens using silver paste as an adhesive (Figure 3a) [22]. The specimen was subjected to both DC and AC measurements using four probe methods: the distance between two outer electrodes for input current was 180 mm, whereas that between two inner electrodes for voltage measurement was 100 mm (Figure 3a). On the other hand, electrical resistance of cubic compressive specimen was measured using a copper wire mesh electrode embedded in the cubic specimens during casting, as shown in Figure 3b. Each cubic compressive specimen was subjected to both DC and AC measurements using two probe methods; the distance between two electrodes was 20 mm, as shown in Figure 3b. The two-probe method was applied for the measurement of electrical resistance of the compressive specimens, unlike tensile specimens, because the dimension of compressive specimen was not large enough for including four electrodes of the four-probe method. In addition, according to Reza et al. [23,24], the two-probe method also successfully measured the change in the electrical resistance even though it obtained higher electrical resistance due to the contact resistance between electrode and specimen. The electrical resistances of three specimens were evaluated in each series.

### 2.2. Test Set-Up and Procedure

**Figure 4:**
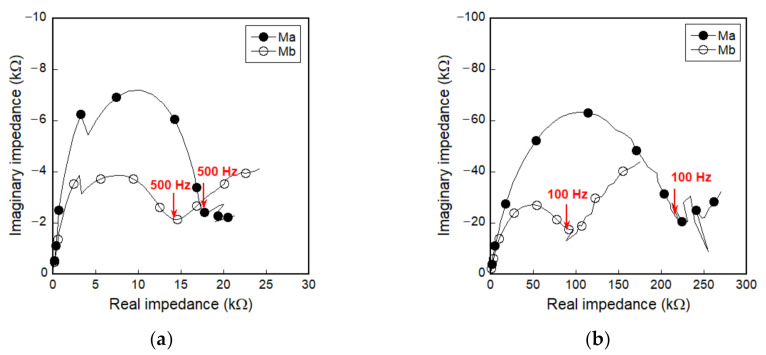
Impedance spectroscopy response of smart ultra-high-performance concretes (smart UHPCs) on alternating current (AC) without load: (**a**) tensile specimens, (**b**) compressive specimens.

**Figure 5:**
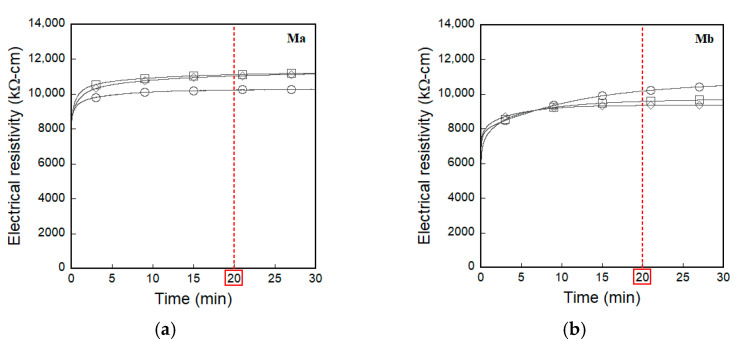
Typical electrical polarization response under direct current measurement without load: (**a**) Ma, (**b**) Mb.

A universal test machine (UTM) was used for both direct tensile and compressive tests. The loading speed and data frequency were 1 mm/min and 5 Hz, respectively. During the direct tensile tests, the applied load was obtained from a 5-ton load cell, while two linear variable differential transformers (LVDTs) were used to measure the tensile elongation of the specimen, as shown in Figure 3a. Their electrical resistance (using DC measurement) was measured using an electrical multimeter (Keysight, 3458 A), while their electrical impedance spectroscopic response (using AC measurement) was measured using an SI1260 impedance/gain-phase analyzer machine (Solatron, 1260 A). The input current for DC measurement was 5 μA. To determine the fixed frequency for AC measurements, the impedance spectroscopy response was obtained from a Nyquist plot with a frequency range of 1 to 10 MHz. In the Nyquist plot, a single arc characterizes the electrical impedance behavior of plain cementitious materials, whereas a two-arc formation represents the response of steel-fiber-reinforced cementitious composites [24]. As seen in Figure 4, 500 Hz and 100 Hz were determined as the fixed frequencies at the cusp, regardless of the matrix type, for tensile and compressive specimens, respectively. For the case of DC measurements, prior to loading, the electrical resistance of specimens was stabilized at least for 20 min without loading, as shown in Figure 5, in order to minimize the effects of electrical polarization. After direct tensile tests, the equivalent number of microcracks was calculated in all the specimens by measuring the total length of all micro-cracks and dividing it by the width of the specimen (50 mm) [4,6]; a Vernier caliper was used to determine the total length. During the tests, the temperature and humidity in the laboratory were 23.05 ± 2.66 °C and 50 ± 17.33%, respectively, under direct tension and 21.0 ± 0.35 °C and 43 ± 3.0%, respectively, under compression.

## 3. Results

The electrical resistance based on the DC measurement was directly measured using a DC multimeter. On the other hand, the electrical resistance based on AC measurement was determined as the value of real impedance (Z’) at the cusp [25]. The electrical resistivity was calculated using Equation (1). The electrical resistivity (*ρ*) is a material property, whereas the cross-sectional area and the distance between the electrodes affect the electrical resistance (*R*).

(1) $$\rho =R\cdot \frac{A}{L}$$

where *ρ* is the electrical resistivity (kΩ·cm), *A* is the cross-sectional area of the specimen (cm^2^), and *L* is the distance between the two inner electrodes (cm).

### 3.1. Electromechanical Response of Smart UHPCs under Direct Tension

The electromechanical responses of smart UHPCs under direct tension were clearly different depending on the electrical current source (DC or AC); however, all smart UHPCs exhibited strain-hardening behavior accompanied by multiple microcracks.

**Figure 6:**
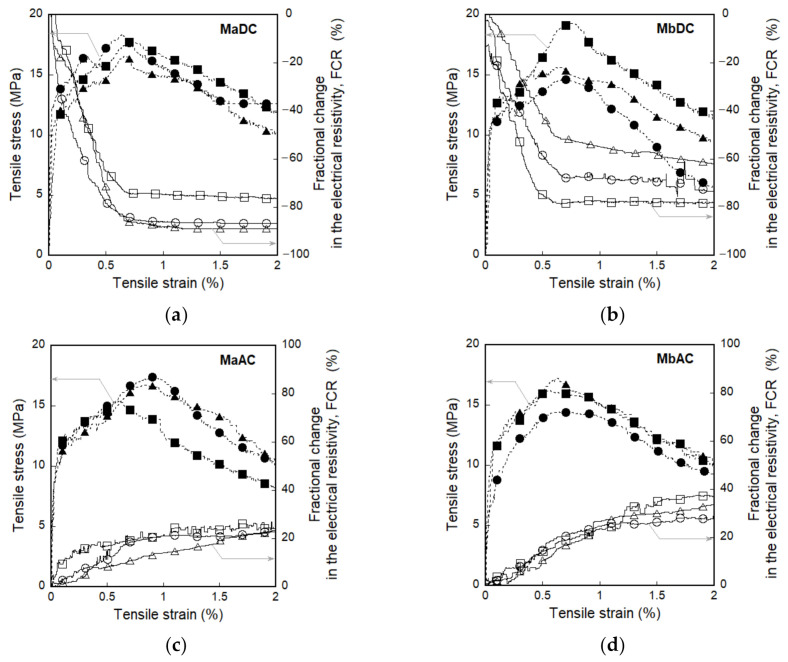
Electromechanical response of smart UHPCs corresponding to the different current sources under direct tension: (**a**) MaDC, (**b**) MbDC, (**c**) MaAC, (**d**) MbAC.

Figure 6 shows the electromechanical response of smart UHPCs under direct tension according to the different current sources. Figure 6a,b show the tensile stress (and fractional change in the electrical resistivity) versus strain of Ma and Mb measured using the DC multimeter (MaDC and MbDC), respectively, while Figure 6c,d display that of Ma and Mb measured using the AC multimeter (MaAC and MbAC), respectively. Table 3 summarizes the values of the mechanical parameters (*ε_cc_*, *ε_pc_*, *σ_cc_*, *σ_pc_*, and *n_cr_*) and electrical parameters (*ρ_i_*, *ρ_cc_*, *ρ_pc_*, Δ*ρ_cc_*, and Δ*ρ_pc_*) of MaDC, MbDC, MaAC, and MbAC.

**Figure 7:**
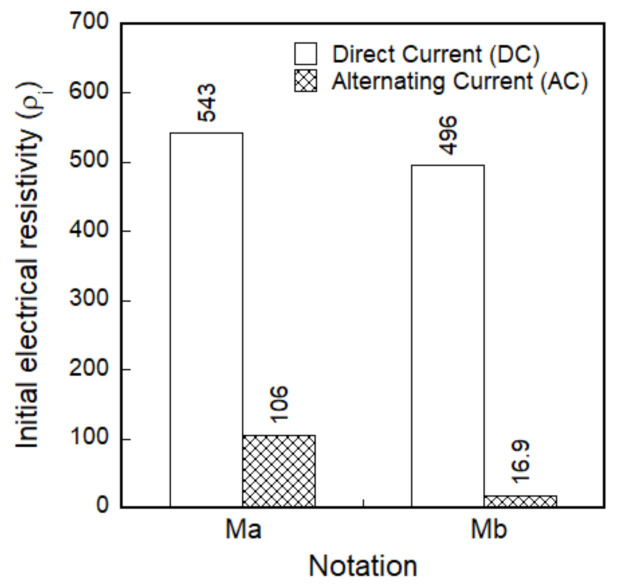
Initial electrical resistivity corresponding to the functional fillers.

Regardless of applied current sources, as seen in Figure 7, the initial electrical resistivities of smart UHPCs are significantly dependent on the functional fillers. The initial electrical resistivities of Ma and Mb under direct tension (*ρ_i_*) were 542.8 and 496.3 kΩ·cm for DC measurement (MaDC and MbDC) and 106.0 and 16.9 kΩ·cm for AC measurement (MaAC and MbAC), respectively. Thus, the electrical resistivities of smart UHPCs containing FSSAs (Mb series) were notably lower than those of Ma series. Moreover, it was noticeable that the electrical resistivities of smart UHPCs measured using a DC multimeter were always higher than those measured using an AC multimeter, regardless of functional fillers. To compare the real parts of electrical impedance measured using an AC multimeter with the resistance measured using a DC multimeter, the fixed frequencies were determined at the point where the imaginary part of the electrical impedance was closest to zero (0), i.e., *R_cusp_* in the Nyquist plot. The real part of the electrical impedance cannot be equal to the resistance measured using a DC multimeter because the real part of the electrical impedance at *R_cusp_* is not exactly zero.

**Figure 8:**
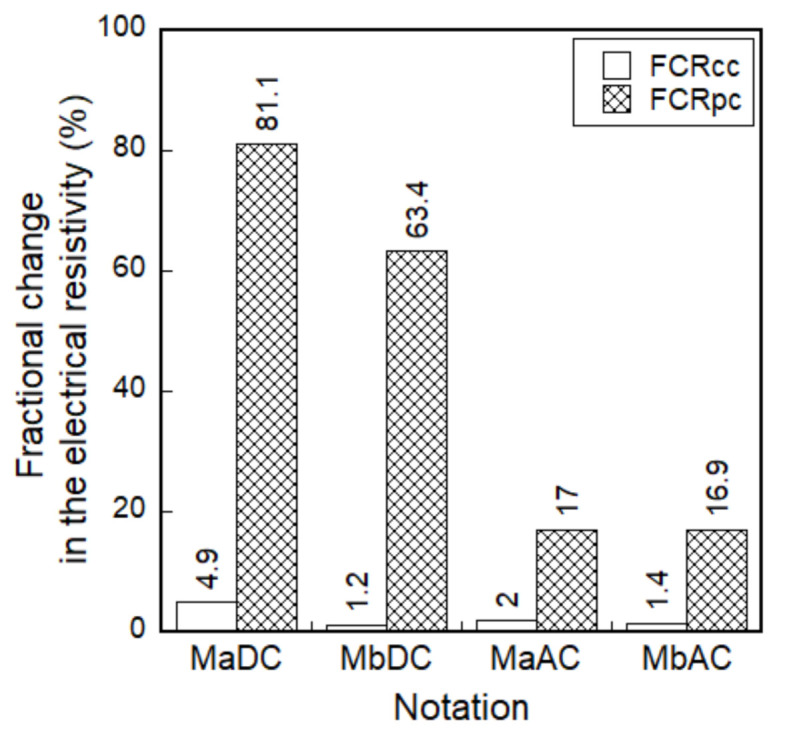
Fractional change in the electrical resistivities (FCR) of smart UHPCs with different current sources and aggregates under direct tension.

The fractional change in the electrical resistivity (FCR) of a specimen under direct tension corresponding to the applied current sources are illustrated in Figure 8. The FCR (=(*ρ_x_*−*ρ_i_*)/*ρ_i_*) is the rate of change in electrical resistivity due to factors such as strain, damage, or stress— *ρ_x_* is the electrical resistivity corresponding to certain value of x coordinate such as strain, damage, stress or time. The fractional change in the electrical resistivity at both first-cracking and postcracking points (*FCR_cc_* and *FCR_pc_*) were calculated to evaluate the strain and damage self-sensing capacities using Equation (2).

(2) $$FCR_{cc}=\left|\frac{\rho_{cc}-\rho_{i}}{\rho_{i}}\right|\times 100=\left|\frac{\Delta \rho_{cc}}{\rho_{i}}\right|\times 100\;FCR_{pc}=\left|\frac{\rho_{pc}-\rho_{cc}}{\rho_{cc}}\right|\times 100=\left|\frac{\Delta \rho_{pc}}{\rho_{cc}}\right|\times 100$$

where, *FCR_cc_* is the FCR at the first-cracking point, *FCR_pc_* is the FCR from the first-cracking point to the postcracking point, *ρ_cc_* is the electrical resistivity at the first-cracking point, *ρ_pc_* is the electrical resistivity at the postcracking point, Δ*ρ_cc_* is the change in the electrical resistivity until first-cracking point, and Δ*ρ_pc_* is the change in the electrical resistivity from the first-cracking point to the postcracking point.

The FCR based on the DC measurement significantly decreased, as the tensile strain increased regardless of functional fillers, as shown in Figure 6a,b, whereas the FCR based on the AC measurement slightly increased as shown in Figure 6c,d. In addition, both *FCR_cc_* and *FCR_pc_* of smart UHPCs based on the DC measurement were significantly higher than those based on the AC measurement. As shown in Figure 8, the *FCR_pc_* of MaDC and MbDC (the DC measurement) values were 81.1% and 63.4%, respectively, while those of MaAC and MbAC (the AC measurement) were 17.0% and 16.9%, respectively.

The use of FSSAs did not generate a significant effect on the FCR values of smart UHPCs, as shown in Figure 8, although the electrical resistivities significantly decreased after adding FSSAs to smart UHPCs. Based on the results obtained using the DC multimeter, the maximum and minimum values of *FCR_cc_* were 4.9% and 1.2%, while those of *FCR_pc_* were 81.1% and 63.4% for MaDC and MbDC series, respectively. The *FCR_cc_* values of MaAC and MbAC were 2.0% and 1.4%, while the *FCR_pc_* values of MaAC and MbAC were 17.0% and 16.9%, respectively.

### 3.2. Electromechanical Responses of Smart UHPCs under Compression

**Figure 9:**
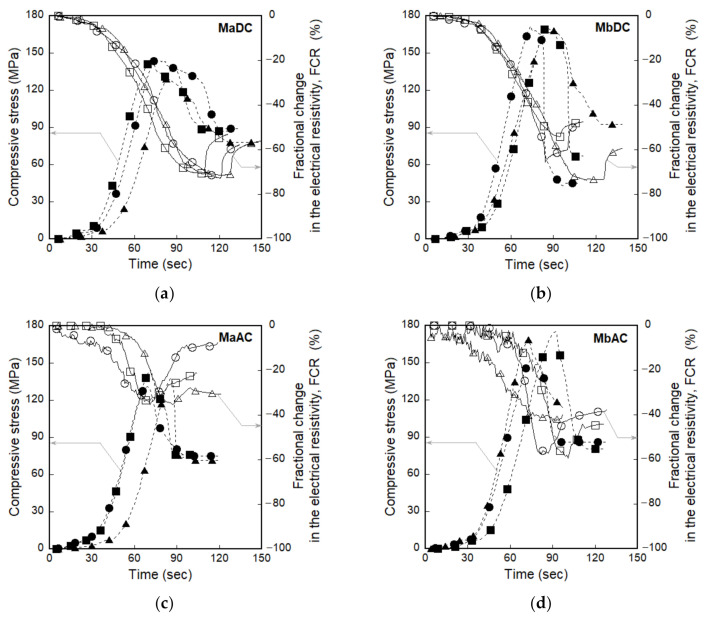
Fractional change in the electrical resistivity (FCR) of smart UHPCs corresponding to the different current sources under compression: (**a**) MaDC, (**b**) MbDC, (**c**) MaAC, (**d**) MbAC.

Figure 9 shows the electromechanical responses of smart UHPCs under compression corresponding to different current sources. Figure 9a,b show the compressive stress (and FCR) versus time curves of MaDC and MbDC, while Figure 9c,d show those of MaAC and MbAC. Table 4 summarizes the peak stress (*σ_p_*) and values of the electrical parameters (*ρ_0_*, *ρ_p_*, and Δ*ρ_p_*) of MaDC, MbDC, MaAC, and MbAC.

As shown in Table 4, the electrical resistivities of smart UHPCs specimens under compression were clearly dependent on the functional fillers, similar to those of the tensile specimens. The initial electrical resistivities (*ρ_0_*) of compressive specimens of MaDC and MbDC were 11352.3 and 10048.6 kΩ·cm, respectively, for DC measurement, while those of MaAC and MbAC were 2584.5 and 1465.6 kΩ·cm, respectively, for AC measurement. Moreover, the electrical resistivities of smart UHPCs measured using a DC multimeter for compressive specimens were always higher than those measured using AC multimeter.

The FCRs based on both DC and AC measurement significantly decreased as the compressive stress increased, as shown in Figure 9. To compare self-stress sensing capacities, *FCR_p_* values were calculated from the curves in Figure 9 using Equation (3).

(3) $$FCR_{p}=\left|\frac{\rho_{p}-\rho_{0}}{\rho_{0}}\right|\times 100=\left|\frac{\Delta \rho_{p}}{\rho_{0}}\right|\times 100$$

where, *FCR_p_* is the FCR at peak stress, *ρ_p_* is the electrical resistivity at peak stress, and Δ*ρ_p_* is the change in the electrical resistivity until peak stress.

The *FCR_p_* values of smart UHPCs based on the DC measurements were higher than those based on the AC measurements; the *FCR_p_* values based on the DC measurement was 47.91% and 47.50% for MaDC and MbDC, respectively, while those based on the AC measurement were 34.43% and 45.63% for MaAC and MbAC, respectively.

**Figure 10:**
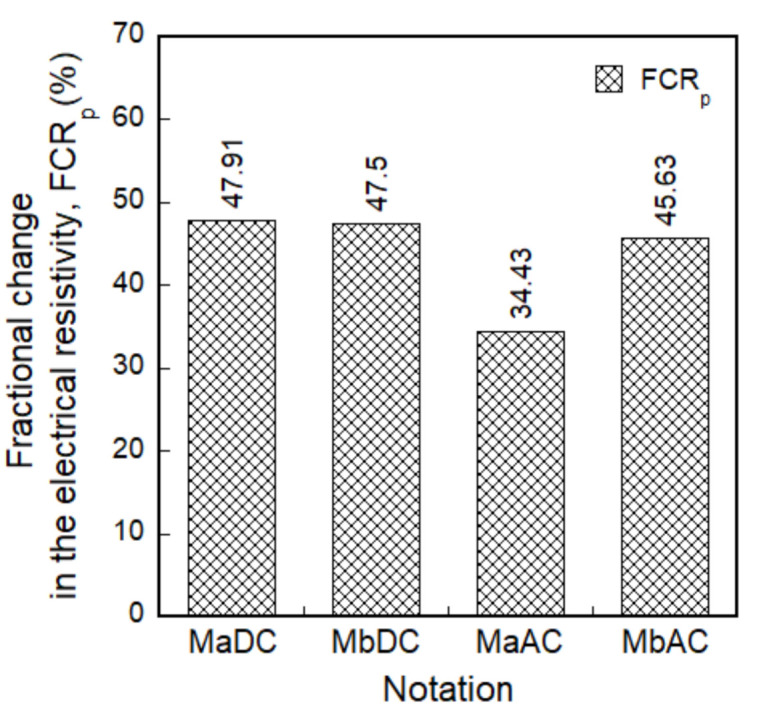
Fractional change in the electrical resistivity (FCR) of smart UHPCs with different current sources and aggregates under compression.

Figure 10 shows the effects of the FSSAs on the FCR values of smart UHPCs. The *FCR_p_* of smart UHPCs based on the DC measurement for Ma (series containing only steel fibers) was similar with Mb (series containing steel fibers and FSSAs), whereas that based on the AC measurement for Mb series was 24.55% higher than that for Ma series.

## 4. Discussion

### 4.1. Electromehcanical Response of Smart UHPCs under Direct Tension

Figure 6 shows the fractional change in the electrical resistivity (FCR) of smart UHPCs under direct tension corresponding to the functional fillers (only steel fibers, Ma; and steel fibers and fine steel slag aggregates, Mb).

The electromechanical responses of smart UHPCs containing steel fibers under direct tension has been reported mainly under DC measurement [2,3,4,6,7]. Le and Kim [21] reported that the reduction in electrical resistivity of SH-SFRCs occurred during fiber-matrix debonding after matrix cracking. Song et al. [2] and Nguyen et al. [3] attributed the reduction in the electrical resistance of SH-SFRCs to the formation of multiple micro-cracks during the strain-hardening region. They explained that the total electrical resistance of the SH-SFRCs could be classified into the electrical resistance of cracked and noncracked parts. The electrical resistance of the cracked part was much lower than that of the noncracked part because steel fibers bridging the microcrack at the cracked part were highly conductive. Thus, the total electrical resistance of SH-SFRC decreased as the number of microcracks increased [2,3,4,6,7].

As the tensile strain of smart UHPCs increased, their electrical resistances measured using a DC multimeter decreased regardless of functional fillers, as shown in Figure 6a,b. The change in the electrical resistivity (Δ*ρ_cc_* and Δ*ρ_pc_*) of MaDC (26.8 and 440.2 kΩ·cm) was higher than that of MbDC (6.2 and 318.47 kΩ·cm). Although the FSSAs were additionally added as functional fillers in the smart UHPCs containing steel fibers, the reason for higher change in the electrical resistivity of smart UHPCs containing only steel fibers is closely related to the fiber bridging under direct tension. The reduction in the electrical resistivity of smart UHPCs containing steel fibers is caused by the electrical current flowing through only the steel fibers connecting the matrix by fiber bridging at the cracked part [2,3,4,6,7]. The change in the electrical resistivity of smart UHPCs containing only steel fibers depends only on the steel fibers, while that containing steel fibers and FSSAs is affected by both steel fibers and FSSAs. The FSSAs in smart UHPCs induces an increase of conductive network between the functional fillers and between the matrix and functional filler, thus the initial electrical resistivity (*ρ_i_*) of smart UHPCs decreases: the *ρ_i_* of Ma (containing only steel fibers) measured using a DC multimeter was 542.8 kΩ·cm, while that of Mb was 496.3 kΩ·cm.

The distance between conductive functional fillers (steel fibers and FSSAs) directly influenced on the tunneling effect [14,15,26], while the number of fibers (*N_f_*) in Equation (4) and the number of contacting fibers (*N_cf_*) in Equation (5), proposed by Xu et al. [27], directly affected the connected electrically conductive networks in smart UHPCs. In this study, both Ma and Mb contained 2 vol.% steel fibers (connected networks between steel fibers was same), the difference in the electrical resistivity between Ma and Mb was dependent upon the distance between functional fillers (FSSAs). The distance between FSSAs (*L_pf_*) was calculated by using the following Equation (6), proposed by Xiao et al. [28], based on the assumption that the fillers are uniformly distributed.

(4) $$N_{f}=\frac{V_{o}\cdot V_{c}}{V_{f}}=\frac{V_{o}\cdot V_{c}}{\pi d_{f}{}^{2}\cdot \frac{l_{f}}{4}}$$

(5) $$N_{cf}=\frac{8\cdot V_{c}\cdot V_{o}\cdot co\;s^{-1}\left(\frac{13.8\times d_{f}}{l_{f}}\right)\sqrt{\frac{1}{V_{o}}}}{{\left(\pi d_{f}\right)}^{2}\times l_{f}}$$

(6) Lpf=dpf×π613×Vpf−13−1

where, *V_o_* and *V_pf_* are the volume content of steel fibers and FSSAs, respectively; *V_c_* and *V_f_* are the volume of composites and a steel fiber, respectively; *l_f_* and *d_f_* are the length and diameter of steel fiber; and *d_pf_* is the diameter of FSSA.

**Figure 11:**
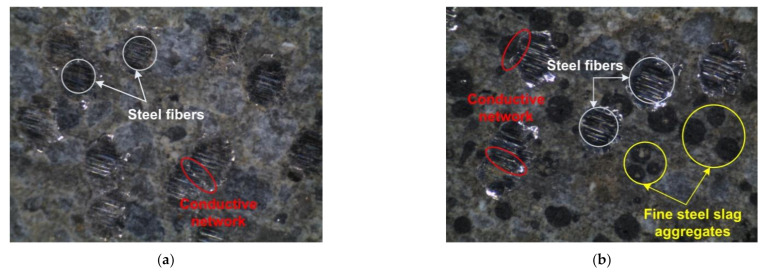
The distribution and conductive network of functional fillers within smart UHPCs: (**a**) Ma series, (**b**) Mb series.

The distribution of functional fillers (steel fibers and FSSAs) in smart UHPCs was observed by using a stereoscopic microscope (Huvits Lusis HC-30MU camera), as shown in Figure 11. Figure 11a,b shows the distribution of fillers in the Ma series containing only steel fibers and that of the Mb series containing steel fibers and FSSAs, respectively. Both steel fibers and FSSAs were uniformly distributed in smart UHPCs, as shown in Figure 11. Moreover, conductive networks of steel fibers and FSSAs were well formed as shown in Figure 11a,b. The *N_f_*, *N_cf_*, and *L_pf_* are summarized in Table 5. The *N_f_* and *N_cf_* of the specimens for tensile tests were calculated as 1179 and 1106 because the length and diameter of steel fibers were 30 and 0.3 mm, respectively. The *L_pf_* in Mb series was calculated as 220.1 μm. Thus, the distance between functional fillers including FSSAs and steel fibers would be lower in the Mb than the Ma series because of the addition of FSSAs in the Mb series.

However, when smart UHPCs under direct tension generated multiple microcracks during tensile stain hardening region, it was questioned whether the electrical resistances of smart UHPCs were mainly affected by the reduced electrical resistance at each cracked part of multiple cracks [2,3,4,6,7] or by the increased distance between functional fillers.

### 4.2. The Self-Sensing Capacity of Smart UHPCs under External Loads Corresponsding to the Different Current Sources

Sensitivity is an important factor evaluating the sensing property of smart UHPCs, and can be characterized by sensitivity coefficient [1]. Although most of the studies evaluated the self-strain- and -damage-sensing capacity of SCMs under direct tension or compression by using gage factors [5,8,29], the stress-sensing capacity of SCMs evaluated by the stress sensitivity coefficient has been more frequently used instead of the gage factor [8,29]. Furthermore, the strain or damage gage factor is also known as strain or damage sensitivity coefficient [1]. Therefore, in this study, sensitivity coefficient was used to compare and analyze strain-, damage-, and stress-sensing capacity. To quantify the self-strain, -damage, and -stress sensing capacity of smart UHPCs, the sensitivity coefficients were calculated using the following Equation (7):

(7) $$SC_{strain}=GF_{strain}=\left|\frac{FCR_{cc}}{\varepsilon_{cc}}\right|\;SC_{damage}=GF_{damage}=\left|\frac{FCR_{pc}}{\varepsilon_{pc}-\varepsilon_{cc}}\right|\;SC_{stress}=\left|\frac{FCR_{p}}{\sigma_{p}}\right|$$

where, *SC_strain_*, *SC_damage_*, and *SC_stress_* are the strain, damage, and stress sensitivity coefficients, respectively, *GF_strain_* and *GF_damage_* are the strain and stress gage factors, respectively.

**Figure 12:**
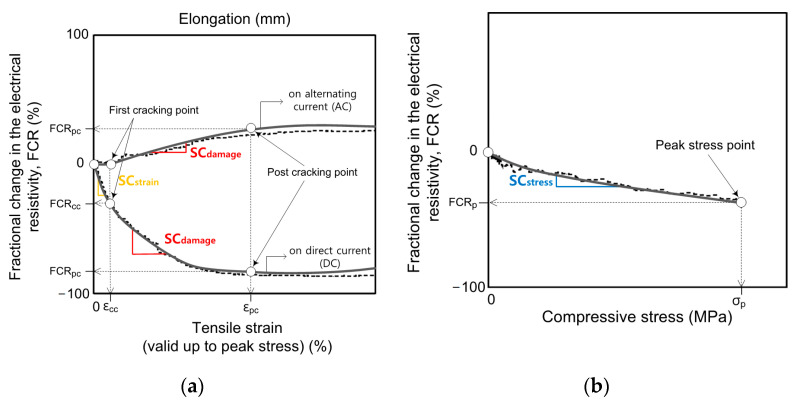
Typical tensile strain or compressive stress until peak stress versus FCR and strain-, damage-, and stress-sensing coefficients of smart UHPCs: (a) under direct tension, (b) under compression.

Figure 12 shows the typical tensile strain (*ε*) or compressive stress (*σ*) until peak stress and FCR of smart UHPCs. Strain and damage sensitivity coefficients (*SC_strain_* and *SC_damage_*) are the ratio between fractional change in the electrical resistivity (FCR) and mechanical strain (*ε*), while a stress sensitivity coefficient (*SC_stress_*) is the ratio of FCR to the mechanical stress (*σ*). *SC_strain_* represents the self-strain sensing capacity of smart UHPCs under direct tension within the elastic range prior to the first cracking, whereas *SC_damage_* represents the self-damage sensing capacity of smart UHPCs under direct tension from first cracking to the post cracking point. Moreover, *SC_stress_* represents the self-stress-sensing capacity of smart UHPCs under compression.

For the smart UHPCs under direct tension, both *SC_strain_* and *SC_damage_* of MaDC were significantly higher than those of MbDC; the *SC_strain_* values of MaDC and MbDC were 188.9% and 42.6%/%, respectively, while the *SC_damage_* values were 128.7% and 96.1%/%, respectively. The *SC_strain_* (67.9%/%) of Ma based on the AC measurement was also higher than that of Mb (46.8%/%), whereas *SC_damage_* of Ma (25.7%/%) and Mb (29.1%/%) values based on the AC measurement were similar. Based on the sensitivity coefficients, both strain and damage self-sensing capacities of Ma (with only steel fibers) were superior to Mb (with both steel fibers and FSSAs). Therefore, DC measurement was more suitable for self-strain- and self-damage-sensing capacities of smart UHPCs than AC measurement.

In comparison with previous research, the *GF_strain_* (188.9%/%) of MaDC in this study was significantly higher than that of SCM containing carbon fiber (59%/%) [30]. The *GF_damage_* of MaDC (129%/%) was significantly higher than that (28.3%/%) of smart UHPCs used by Kim et al. [6], whereas the *GF_strain_* (188.9%/%) of MaDC was lower than that (433%/%) of smart UHPCs used by Kim et al. [6]. On the other hand, from the AC measurement, the *GF_strain_* (67.97%/%) of MaAC was lower than that (247%/%) of SCMs containing carbon blacks but higher than that (24%/%) of the SCMs containing PVA fibers.

Although the *FCR_p_* values of MaDC and MbDC were similar, the *SC_stress_* (0.33%/MPa) of MaDC was higher than that (0.28%/MPa) of MbDC. However, the *SC_stress_* of MbAC (0.27%/MPa) was slightly higher than that of MaAC (0.25%/MPa), although the *FCR_p_* of MbAC (45.63%) was much higher than that of MaAC (34.43%). Thus, the effect of adding FSSAs on the self-stress-sensing capacities of smart UHPCs was greater under DC measurement than under AC measurement.

Regarding the *SC_stress_*, MaDC produced the highest *SC_stress_* (0.33%/MPa) from the DC measurement while MbAC did the highest one (0.27%/MPa) from the AC measurement. The (0.28%/MPa) of SCMs containing carbon black from DC measurement, reported by Monteiro et al. [29], was slightly lower than that (0.33%/MPa) of MaDC, while the *SC_stress_* (0.41%/MPa) of the smart UHPCs containing only 1.0% FSSAs from AC measurement [8] was higher than that (0.27%/MPa) of MbAC.

### 4.3. Effects of Different Current Sources (DC or AC) on the Electromechanical Response of Smart UHPCs under External Loads

As the tensile strain of smart UHPCs increased, their electrical resistances measured from DC multimeter decreased regardless of functional fillers, as shown in Figure 6a,b. However, the electrical resistances of smart UHPCs measured using AC multimeter increased as their tensile strain increased, as shown in Figure 6c,d. The self-sensing mechanisms of smart UHPCs based on the AC measurement commonly include tunneling effects and conductive networks. In other words, the change in the electrical resistance (or response) of smart UHPCs under AC measurement was primarily due to the tunneling effects between two conductive particles and electrically connected (conductive) networks, including continuously connected pores and connected steel or carbon fibers [11,13]. However, under DC measurements, such changes were mostly dependent on the fiber crack bridging effects at cracked parts, as explained before. Thus, the electrical resistances of smart UHPCs under direct tension, when they were measured from AC multimeter, increased because of longer distance between conductive functional fillers (FSSAs) and the stretched conductive network of steel fibers in the composites.

**Figure 13:**
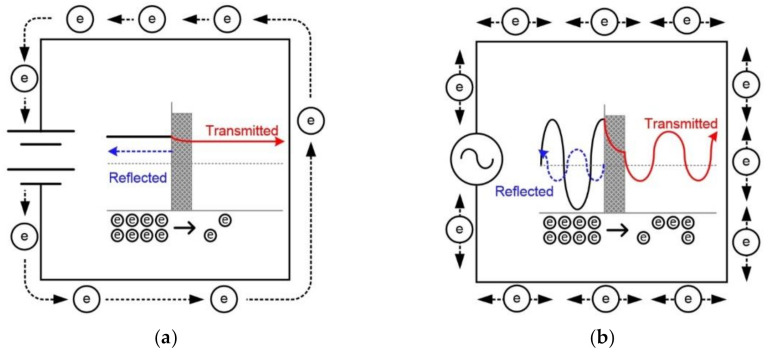
Theory of movement of electrons based on the tunneling effects [31,32]: (**a**) under direct current, (**b**) under alternating current.

The basis of different self-sensing mechanisms corresponding to different electrical current source would be the different relationships between charge mobility and tunneling effect. Figure 13 illustrates the movement of electrons under DC or AC current. Electrons move from the negative to the positive pole under DC measurement, whereas under AC measurement electrons remain static and vibrate in their current positions [31]. The tunneling effect refers to a phenomenon in which electrons pass through an electromagnetic barrier because the kinetic energy of the electrons is greater than that of the electromagnetic barrier [32]. Thus, the tunneling effect under AC measurement would be higher than that under DC measurement. Moreover, difference in electrical current penetration, according to DC and AC measurements, would also generate the different electrical resistance. The magnetic field created by DC generally penetrates the entire cross-section of the object, whereas that created by AC is concentrated in a thin layer on the object, that called “skin effect” [33]. Consequently, the initial electrical resistivity of specimens measured from AC multimeter was significantly lower than that of specimens measured from DC multimeter. Furthermore, penetration depth is also affected by the frequency—as the frequency increases, the penetration depth decreases [34]. The fixed frequency, in this study, for tensile specimen was 500 Hz, whereas that for compressive specimen was 100 Hz. Thus, the penetration depth in tensile specimens with high frequency was smaller than that in compressive specimens with low frequency. Consequently, the initial electrical resistivity of a compressive specimen with high penetration depth was higher than that of a tensile specimen. The electrical resistance of smart UHPCs under DC measurement would primarily depend on fiber crack bridging, while that under AC measurement would primarily depend on the tunneling effect. Hence, as the tensile strain increased with an increasing number of multiple micro cracks, the electrical resistance of smart UHPCs under DC measurement decreased, owing to fiber crack-bridging effects, whereas that under AC measurement increased owing to tunneling effects.

**Figure 14:**
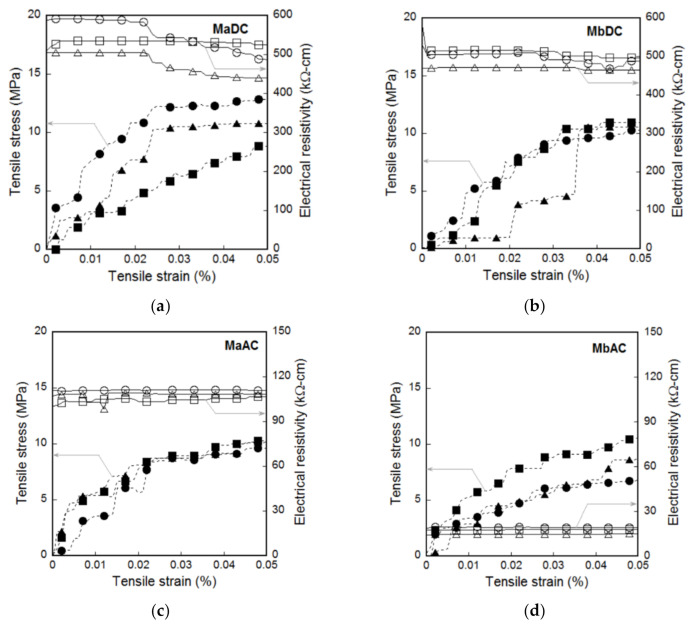
Electrical resistivity responses until first cracking point of smart UHPCs corresponding to the different current sources under direct tension: (**a**) MaDC, (**b**) MbDC, (**c**) MaAC, (**d**) MbAC.

In addition, self-strain-sensing capacity of smart UHPCs until *σ_cc_* (first-cracking strength point) was notably different corresponding to the applied electrical current source. As shown in Figure 14, the electrical resistivity of smart UHPCs under DC measurement slightly changed prior to the first cracking strength point, but it significantly decreased after that point. Even though the tensile strain of smart UHPCs increased gradually prior to the first cracking strength point, the electrical resistance of smart UHPCs changed slightly prior to first cracking. This was because the electrical current mostly flowed through the mortar matrix under DC measurement, which mainly depends on fiber crack bridging. However, after first cracking, the electrical current started to flow through the highly conductive steel fibers at the cracked section. Therefore, the electrical resistances of smart UHPCs started to decrease noticeably from the first cracking point to post cracking point. On the other hand, the electrical resistances of smart UHPCs changed slightly under AC measurement because the electromechanical responses of smart UHPCs measured using the AC multimeter were primarily dependent on the tunneling effect. The tunneling effect was nearly constant prior to the first cracking point, as the tensile strain was very small. Thus, DC measurements would be more suitable than AC measurements for the tensile self-strain sensing of smart UHPCs.

**Figure 15:**
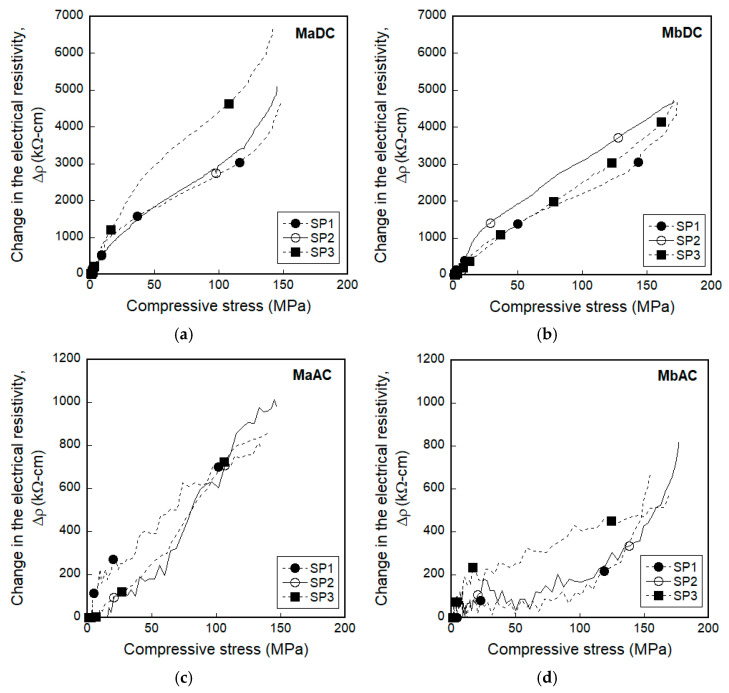
Electrical resistivity responses of smart UHPCs under compression: (**a**) MaDC, (**b**) MbDC, (**c**) MaAC, (**d**) MbAC.

In general, the changes in the electrical resistivities increased linearly as the applied compressive stress increased, as shown in Figure 15. The linear relationship would be utilized to measure the amount of applied compressive stress based on the measured electrical resistivity. Moreover, the electrical resistivities of smart UHPCs under compression decreased as the compressive stress increased, regardless of the applied electrical current sources, because the electromechanical responses of smart UHPCs under compression were only dependent on the tunneling effect.

## 5. Conclusions

This study investigated the electromechanical responses of smart UHPCs corresponding to the different current sources (DC or AC) to clarify self-strain-, -damage-, and -stress-sensing mechanisms, and consequently to suggest a suitable measurement method of electrical response according to the self-sensing purpose.

- The self-sensing mechanism from DC measurement would be mainly dependent on fiber crack bridging, whereas that from AC measurement was primarily dependent on the tunneling effect.
- The electrical resistivities, from both DC and AC measurements, of smart UHPCs under compression clearly decreased as the applied stress increased, regardless of the types of aggregates, because the electrical responses of smart UHPCs under compression were primarily dependent upon the tunneling effect.
- Regarding the stress self-sensing capacities of smart UHPCs under compression, the self-sensing capacities of smart UHPCs from DC measurement were moderately higher than that from AC measurement: the *SC_stress_* of MaDC and MaAC were 0.33% and 0.25%/MPa, respectively, while those of MbDC and MbAC were 0.28% and 0.27%/MPa, respectively.
- Under tension, as the tensile strain and the number of multiple microcracks of smart UHPCs increased, the electrical resistances from DC measurement significantly decreased, whereas those from AC measurement slightly increased owing, to different self-sensing mechanism.
- Regarding the self-strain- and -damage-sensing capacities of smart UHPCs under tension, the self-sensing capacities of smart UHPCs from DC measurement were significantly higher those from AC measurement: the *SC_strain_* values of MaDC and MaAC were 188.9% and 67.9%/%, respectively, while *SC_damage_* values of MaDC and MaAC were 128.7% and 25.7%/%, respectively.

In future research, we intend to investigate the effects of different (embedded or attached) electrodes on the electromechanical responses of smart UHPCs corresponding to different loading conditions. Moreover, it is necessary to further investigate the electromechanical response of smart UHPCs containing different aggregates as functional fillers.

## Figures and Tables

**Table 1:** Composition of matrix by weight ratio.

Notation	Cement (Type 1)	Silica Sand	Fine Steel Slag Aggregate	Silica Fume	Silica Powder	Water	Super-Plasticizer *	*f′_ck_* (MPa)
Ma	1.0	1.00	-	0.15	0.25	0.20	0.042	178
Mb	1.0	0.50	0.50	0.15	0.25	0.20	0.042	179

* Superplasticizer contained 30% solid content.

**Table 2:** Properties of functional fillers.

Type	Diameter (μm)	Length (mm)	Density (g/cm^3^)	Tensile Stregnth (MPa)	Elastic Modulus (GPa)
Long smooth steel fibers	300	30	7.9	2447	200
Short smooth steel fibers	200	6	7.9	2104	200
Fine steel slag aggregate	<390	-	-	-	-

**Table 3:** Electro-tensile parameters of smart UHPCs.

Notation	Spe.	Strain (%)		Stress (MPa)		Crack	Electrical Resistivity (kΩ·cm)			Change in the Electrical Resistivity (kΩ·cm)	
		*ε_cc_*	*ε_pc_*	*σ_cc_*	*σ_pc_*	*n_cr_*	*ρ_i_*	*ρ_cc_*	*ρ_pc_*	Δ*ρ_cc_*	Δ*ρ_pc_*
Ma DC	SP1	0.024	0.63	12.09	18.35	23.4	588.8	550.9	100.9	37.9	487.9
	SP2	0.045	0.70	8.00	17.72	22.1	534.3	530.2	131.8	4.1	402.5
	SP3	0.027	0.66	10.38	16.56	20.4	505.3	466.9	75.2	38.4	430.1
	Aver.	0.032	0.66	10.16	17.54	22.0	542.8	516.0	102.6	26.8	440.2
	STD	0.009	0.03	1.68	0.74	1.2	34.6	35.7	23.1	16.1	35.6
Mb DC	SP1	0.029	0.68	9.37	14.64	19.2	503.1	492.0	188.3	11.1	314.8
	SP2	0.030	0.74	8.76	19.28	19.7	515.0	512.8	116.7	2.2	398.3
	SP3	0.029	0.65	4.34	15.63	18.6	470.9	465.8	227.5	5.1	243.4
	Aver.	0.029	0.69	7.49	16.52	19.2	496.3	490.2	177.5	6.2	318.47
	STD	0.000	0.04	2.24	2.00	0.4	18.6	19.2	45.9	3.7	63.3
Ma AC	SP1	0.024	0.63	10.55	19.28	27.1	110.8	111.1	133.5	−0.3	−22.4
	SP2	0.029	0.66	10.55	16.54	25.7	100.4	104.5	118.6	−4.1	−14.1
	SP3	0.029	0.89	8.73	17.39	23.3	106.9	108.5	120.2	−1.6	−11.7
	Aver.	0.027	0.73	9.94	17.74	25.4	106.0	108.0	124.1	−2.0	−16.1
	STD	0.002	0.12	0.86	1.15	1.6	4.3	2.7	6.7	1.6	4.6
Mb AC	SP1	0.030	0.60	5.71	17.22	19.5	19.0	19.3	22.8	−0.3	−3.5
	SP2	0.029	0.59	8.46	18.40	19.1	17.6	17.8	20.3	−0.2	−2.5
	SP3	0.029	0.65	6.07	17.36	18.9	14.2	14.4	16.4	−0.2	−2.0
	Aver.	0.029	0.61	6.75	17.66	19.2	16.9	17.2	19.8	−0.2	−2.7
	STD	0.000	0.03	1.22	0.53	0.2	2.0	2.0	2.6	0.0	0.6

*n_cr_*: number of microcracks; STD: standard derivation.

**Table 4:** Electro-compressive parameters of smart UHPCs.

Notation	Spe. No.	Peak Stress (MPa)	Initial Electrical Resistivity (kΩ·cm)	Electrical Resistivity at Peak Stress (kΩ·cm)	Change in the Electrical Resistivity (kΩ·cm)
		*σ_p_*	*ρ_0_*	*ρ_p_*	Δ*ρ_p_*
Ma DC	SP1	147.97	11544.9	6902.5	4642.4
	SP2	145.15	10992.6	5957.3	5035.3
	SP3	142.09	11519.5	4872.4	6647.1
	Aver.	145.07	11352.3	5910.7	5441.6
	STD	2.40	254.6	829.4	867.4
Mb DC	SP1	173.52	11069.9	6323.2	4746.7
	SP2	171.16	9478.3	4759.4	4718.9
	SP3	171.16	9597.4	4814.9	4782.5
	Aver.	171.95	10048.6	5299.2	4749.4
	STD	1.11	723.9	724.5	26.0
Ma AC	SP1	134.08	2430.5	1620.6	809.9
	SP2	146.56	2878.2	1866.2	1012.0
	SP3	139.97	2444.9	1593.9	851.0
	Aver.	140.20	2584.5	1693.6	891.0
	STD	5.10	207.8	122.6	87.2
Mb AC	SP1	155.15	1481.5	847.2	634.3
	SP2	176.82	1482.1	685.6	796.5
	SP3	170.22	1433.1	855.2	577.9
	Aver.	167.40	1465.6	796.0	669.6
	STD	9.07	23.0	78.1	92.7

**Table 5:** The number of fibers (*N_f_*), the number of contacting fibers (*N_cf_*), and distance between fine steel slag aggregates (*L_pf_*) of the smart UHPCs under tension.

Loading Condition	Matrix Notation	The Number of Steel Fibers, *N_f_*	The Number of Initial Contacting Steel Fibers, *N_cf_*	Initial Distance between FSSAs, *L_pf_* (μm)
Tension	Ma	1179	1106	-
	Mb	1179	1106	220.1

## Data Availability

Not applicable.
